# Supplementary material for: Identification of photoperiod-induced specific miRNAs in the adrenal glands of Sunite sheep (Ovis aries)
Source: Front Vet Sci. 2022 Jul 22;9:888207. doi: 10.3389/fvets.2022.888207 (PMC9354845; doi:10.3389/fvets.2022.888207)
Supplement: Supplementary file 1 [file Data_Sheet_1.ZIP › Supplementary file/miRDeep2 file.zip!/miRDeep2 file/Novel_Structure/SP42A2/Novel_435.pdf]

Provisional ID	: NC_019459.2_5309	
Score total	:	2.4
Score for star read(s)	:	-1.3
Score for read counts	:	0
Score for mfe	:	2.1
Score for randfold	:	1.6
Score for cons. seed	:	
Total read count	:	86
Mature read count	:	83
Loop read count	:	0
Star read count	:	3

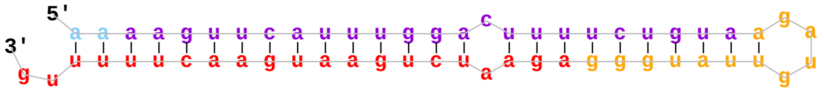

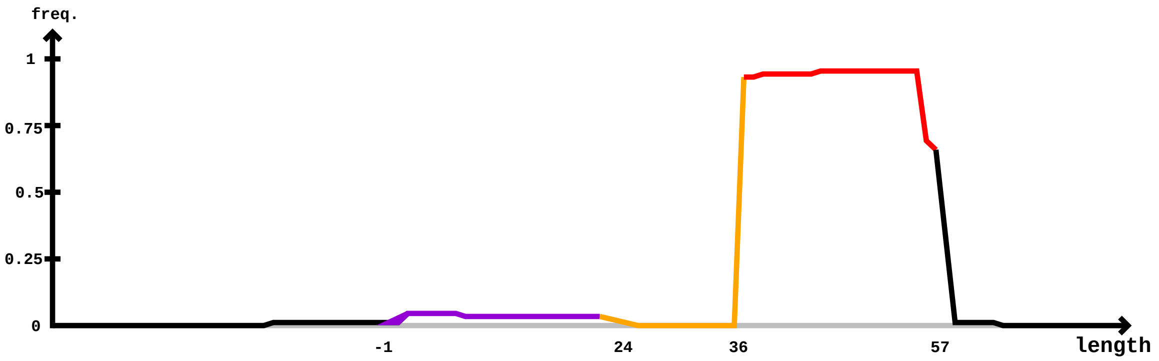

Star Mature

```
5'- aguuuguuuauucuauauauauuggauuggucaaaaaguucauuuggacuuuucuguaagauguuauggg agaaucugaaugaacuuuuuggccaaucccaauaagcagu   -3'   obs
    aguuuguuuauucuauauauauuggauuggucaaaaaguucauuuggacuuuucuguaagauguuaugggagaaucugaaugaacuuuuuggccaaucccaauaagcagu         exp
    ...(((((((((...........(((((((((((((((((((((((((.((((((((((....)))))))))).))))))))))))))))))))))))).))))))))).   reads   mm   sample
    .....................uuggauuggCcaaaaaguuc.....................................................................   1       1    seq
    ...................................aaguucauuuggGcuuuucugua....................................................   3       1    seq
    ......................................................................aAaaucugaaugaacuuuu.....................   23      1    seq
    ......................................................................agaaucugaaugaacuuuuu....................   2       0    seq
    ......................................................................aAaaucugaaugaacuuuuug...................   57      1    seq
    ........................................................................aaucugaaugaacuuuuu....................   1       0    seq
    ..............................................................................aaCgaacuuuuuggccaau.............   1       1    seq
```
